# Supplementary material for: EA.hy926 Cells and HUVECs Share Similar Senescence Phenotypes but Respond Differently to the Senolytic Drug ABT-263
Source: Cells. 2022 Jun 21;11(13):1992. doi: 10.3390/cells11131992 (PMC9266052; doi:10.3390/cells11131992)
Supplement: Supplementary file 1 [file cells-11-01992-s001.zip › cells-1751426-supplementary.pptx]

## Slide 1
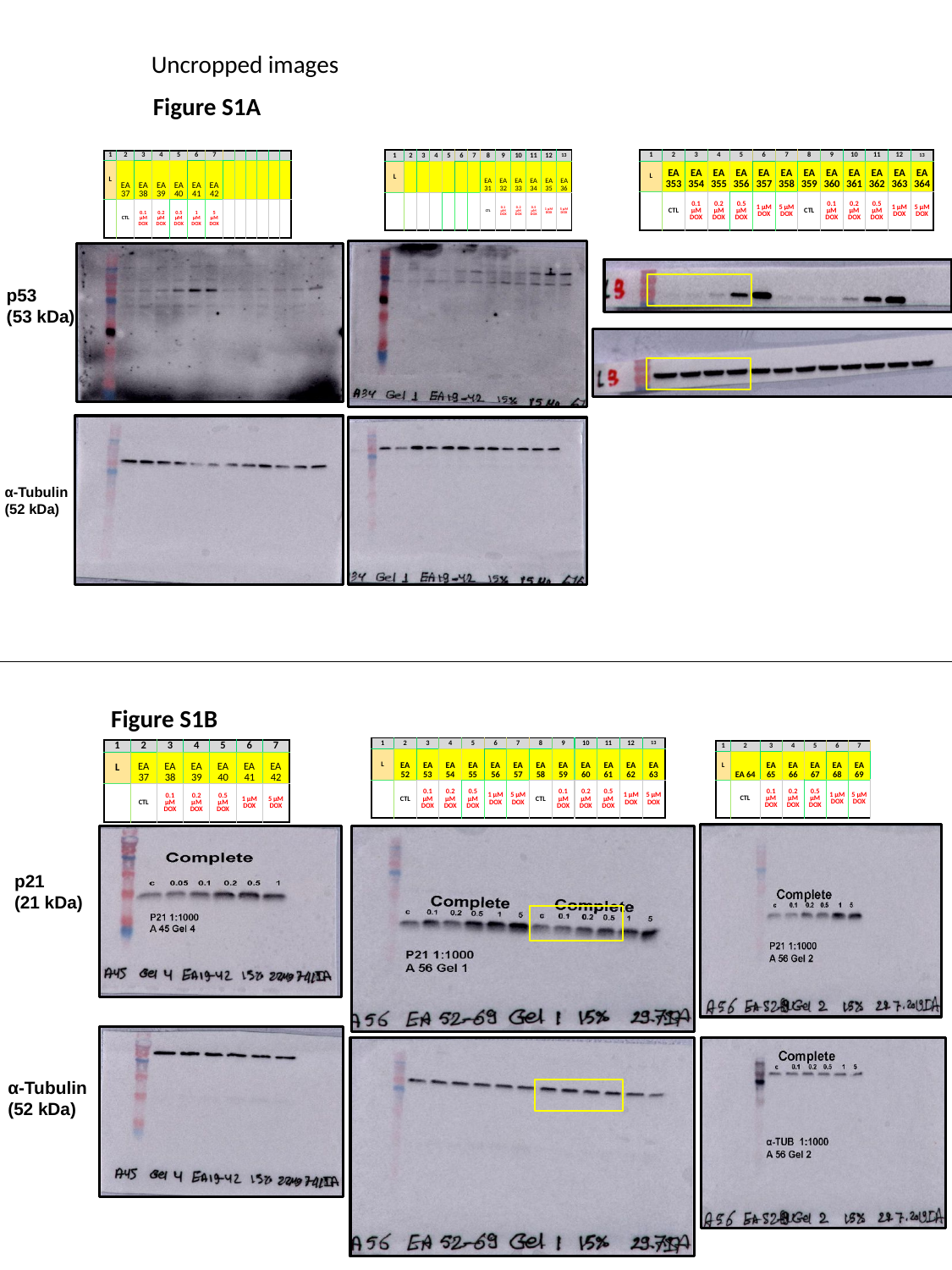

Uncropped images
Figure S1A
| 1 | 2 | 3 | 4 | 5 | 6 | 7 | 8 | 9 | 10 | 11 | 12 | 13 |
| --- | --- | --- | --- | --- | --- | --- | --- | --- | --- | --- | --- | --- |
| L | EA 353 | EA 354 | EA 355 | EA 356 | EA 357 | EA 358 | EA 359 | EA 360 | EA 361 | EA 362 | EA 363 | EA 364 |
| | CTL | 0.1 µM DOX | 0.2 µM DOX | 0.5 µM DOX | 1 µM DOX | 5 µM DOX | CTL | 0.1 µM DOX | 0.2 µM DOX | 0.5 µM DOX | 1 µM DOX | 5 µM DOX |
| 1 | 2 | 3 | 4 | 5 | 6 | 7 | 8 | 9 | 10 | 11 | 12 | 13 |
| --- | --- | --- | --- | --- | --- | --- | --- | --- | --- | --- | --- | --- |
| L | | | | | | | EA 31 | EA 32 | EA 33 | EA 34 | EA 35 | EA 36 |
| | | | | | | | CTL | 0.1 µM DOX | 0.2 µM DOX | 0.5 µM DOX | 1 µM DOX | 5 µM DOX |
| 1 | 2 | 3 | 4 | 5 | 6 | 7 | | | | | | |
| --- | --- | --- | --- | --- | --- | --- | --- | --- | --- | --- | --- | --- |
| L | EA 37 | EA 38 | EA 39 | EA 40 | EA 41 | EA 42 | | | | | | |
| | CTL | 0.1 µM DOX | 0.2 µM DOX | 0.5 µM DOX | 1 µM DOX | 5 µM DOX | | | | | | |
p53
(53 kDa)
α-Tubulin
(52 kDa)
Figure S1B
| 1 | 2 | 3 | 4 | 5 | 6 | 7 | 8 | 9 | 10 | 11 | 12 | 13 |
| --- | --- | --- | --- | --- | --- | --- | --- | --- | --- | --- | --- | --- |
| L | EA 52 | EA 53 | EA 54 | EA 55 | EA 56 | EA 57 | EA 58 | EA 59 | EA 60 | EA 61 | EA 62 | EA 63 |
| | CTL | 0.1 µM DOX | 0.2 µM DOX | 0.5 µM DOX | 1 µM DOX | 5 µM DOX | CTL | 0.1 µM DOX | 0.2 µM DOX | 0.5 µM DOX | 1 µM DOX | 5 µM DOX |
| 1 | 2 | 3 | 4 | 5 | 6 | 7 |
| --- | --- | --- | --- | --- | --- | --- |
| L | EA 37 | EA 38 | EA 39 | EA 40 | EA 41 | EA 42 |
| | CTL | 0.1 µM DOX | 0.2 µM DOX | 0.5 µM DOX | 1 µM DOX | 5 µM DOX |
| 1 | 2 | 3 | 4 | 5 | 6 | 7 |
| --- | --- | --- | --- | --- | --- | --- |
| L | EA 64 | EA 65 | EA 66 | EA 67 | EA 68 | EA 69 |
| | CTL | 0.1 µM DOX | 0.2 µM DOX | 0.5 µM DOX | 1 µM DOX | 5 µM DOX |
p21
(21 kDa)
α-Tubulin
(52 kDa)

## Slide 2
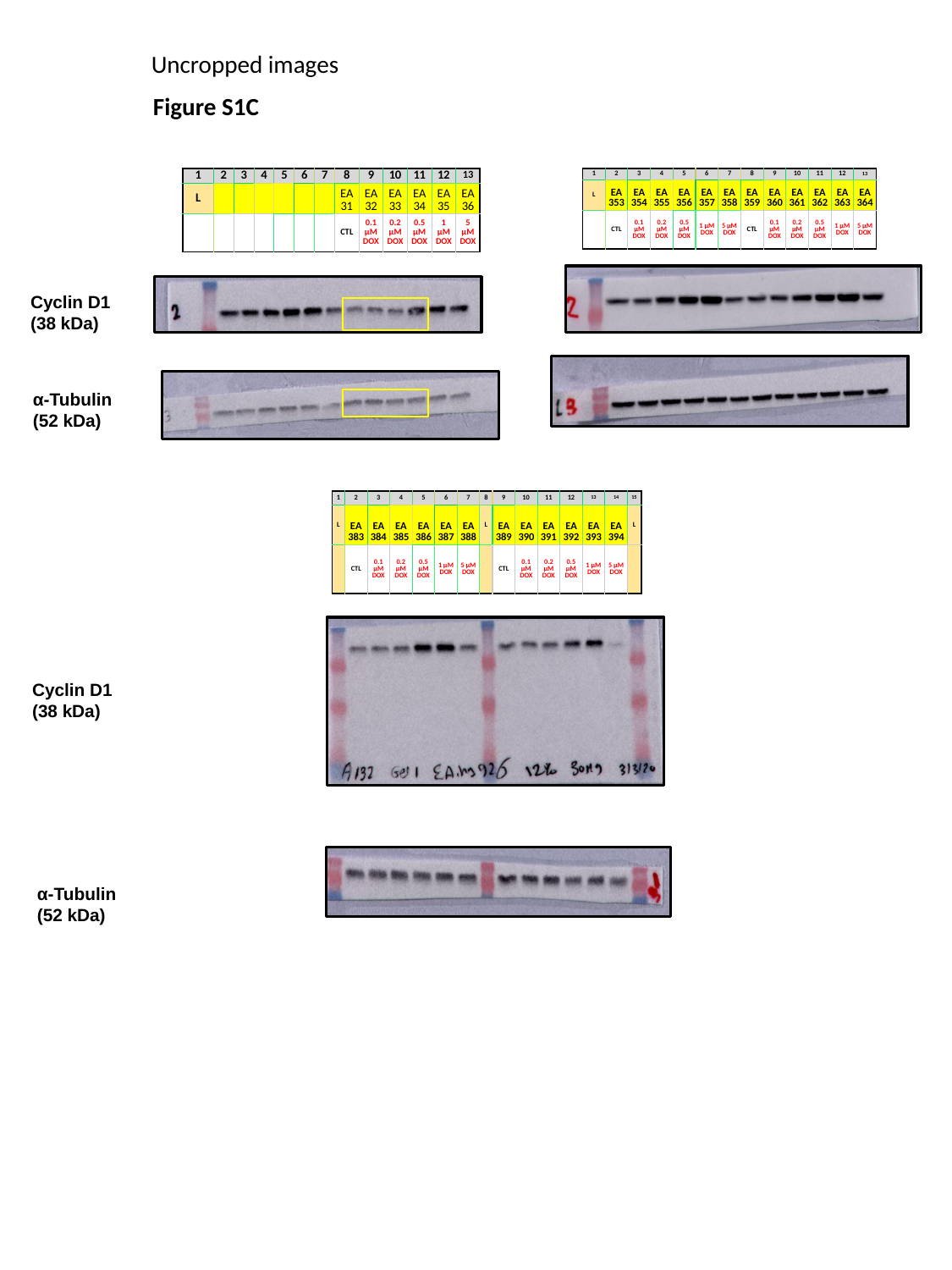

Uncropped images
Figure S1C
| 1 | 2 | 3 | 4 | 5 | 6 | 7 | 8 | 9 | 10 | 11 | 12 | 13 |
| --- | --- | --- | --- | --- | --- | --- | --- | --- | --- | --- | --- | --- |
| L | | | | | | | EA 31 | EA 32 | EA 33 | EA 34 | EA 35 | EA 36 |
| | | | | | | | CTL | 0.1 µM DOX | 0.2 µM DOX | 0.5 µM DOX | 1 µM DOX | 5 µM DOX |
| 1 | 2 | 3 | 4 | 5 | 6 | 7 | 8 | 9 | 10 | 11 | 12 | 13 |
| --- | --- | --- | --- | --- | --- | --- | --- | --- | --- | --- | --- | --- |
| L | EA 353 | EA 354 | EA 355 | EA 356 | EA 357 | EA 358 | EA 359 | EA 360 | EA 361 | EA 362 | EA 363 | EA 364 |
| | CTL | 0.1 µM DOX | 0.2 µM DOX | 0.5 µM DOX | 1 µM DOX | 5 µM DOX | CTL | 0.1 µM DOX | 0.2 µM DOX | 0.5 µM DOX | 1 µM DOX | 5 µM DOX |
Cyclin D1
(38 kDa)
α-Tubulin
(52 kDa)
| 1 | 2 | 3 | 4 | 5 | 6 | 7 | 8 | 9 | 10 | 11 | 12 | 13 | 14 | 15 |
| --- | --- | --- | --- | --- | --- | --- | --- | --- | --- | --- | --- | --- | --- | --- |
| L | EA 383 | EA 384 | EA 385 | EA 386 | EA 387 | EA 388 | L | EA 389 | EA 390 | EA 391 | EA 392 | EA 393 | EA 394 | L |
| | CTL | 0.1 µM DOX | 0.2 µM DOX | 0.5 µM DOX | 1 µM DOX | 5 µM DOX | | CTL | 0.1 µM DOX | 0.2 µM DOX | 0.5 µM DOX | 1 µM DOX | 5 µM DOX | |
Cyclin D1
(38 kDa)
α-Tubulin
(52 kDa)

## Slide 3
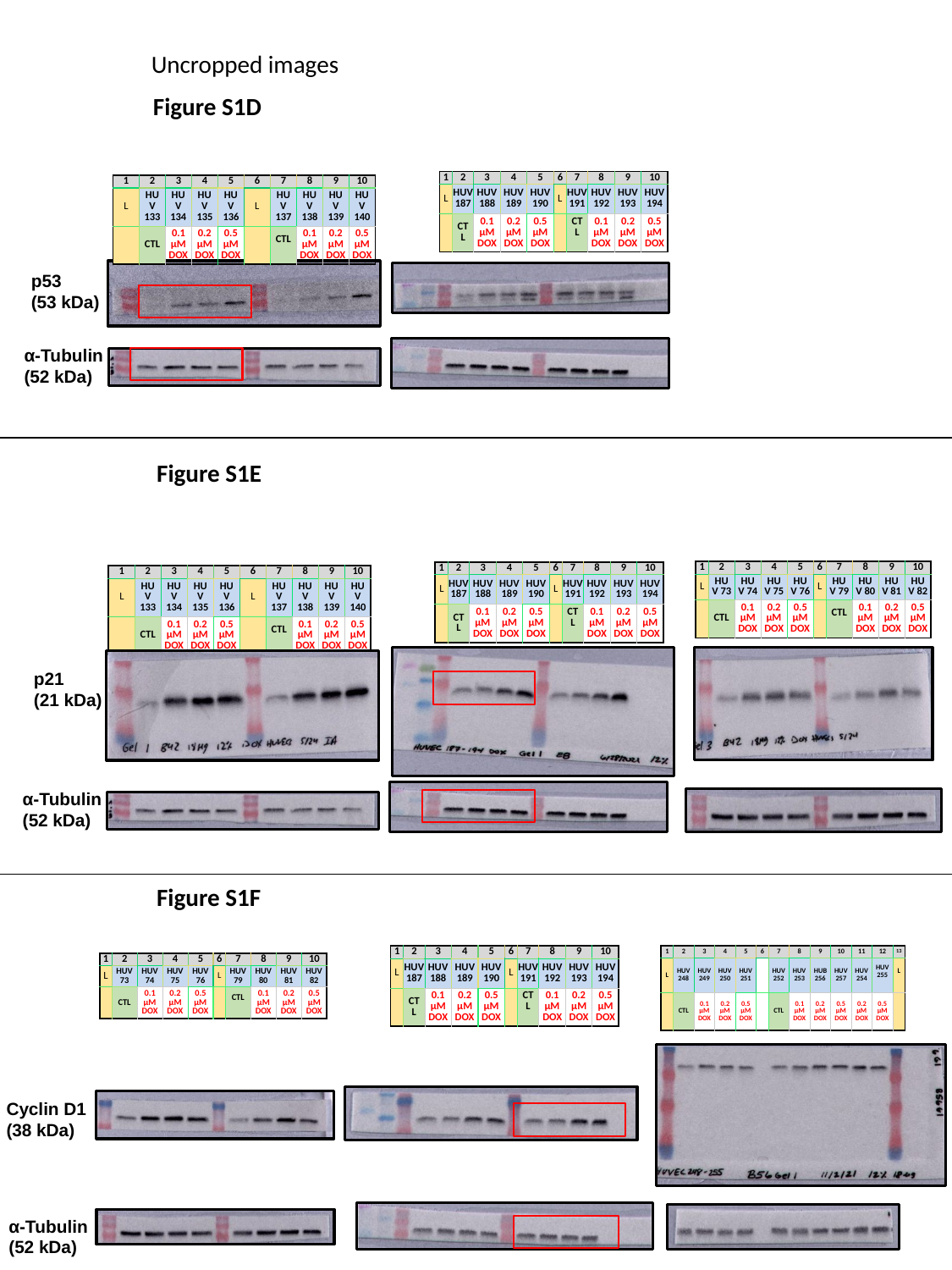

Uncropped images
Figure S1D
| 1 | 2 | 3 | 4 | 5 | 6 | 7 | 8 | 9 | 10 |
| --- | --- | --- | --- | --- | --- | --- | --- | --- | --- |
| L | HUV 187 | HUV 188 | HUV 189 | HUV 190 | L | HUV 191 | HUV 192 | HUV 193 | HUV 194 |
| | CTL | 0.1 µM DOX | 0.2 µM DOX | 0.5 µM DOX | | CTL | 0.1 µM DOX | 0.2 µM DOX | 0.5 µM DOX |
| 1 | 2 | 3 | 4 | 5 | 6 | 7 | 8 | 9 | 10 |
| --- | --- | --- | --- | --- | --- | --- | --- | --- | --- |
| L | HUV 133 | HUV 134 | HUV 135 | HUV 136 | L | HUV 137 | HUV 138 | HUV 139 | HUV 140 |
| | CTL | 0.1 µM DOX | 0.2 µM DOX | 0.5 µM DOX | | CTL | 0.1 µM DOX | 0.2 µM DOX | 0.5 µM DOX |
p53
(53 kDa)
α-Tubulin
(52 kDa)
Figure S1E
| 1 | 2 | 3 | 4 | 5 | 6 | 7 | 8 | 9 | 10 |
| --- | --- | --- | --- | --- | --- | --- | --- | --- | --- |
| L | HUV 73 | HUV 74 | HUV 75 | HUV 76 | L | HUV 79 | HUV 80 | HUV 81 | HUV 82 |
| | CTL | 0.1 µM DOX | 0.2 µM DOX | 0.5 µM DOX | | CTL | 0.1 µM DOX | 0.2 µM DOX | 0.5 µM DOX |
| 1 | 2 | 3 | 4 | 5 | 6 | 7 | 8 | 9 | 10 |
| --- | --- | --- | --- | --- | --- | --- | --- | --- | --- |
| L | HUV 187 | HUV 188 | HUV 189 | HUV 190 | L | HUV 191 | HUV 192 | HUV 193 | HUV 194 |
| | CTL | 0.1 µM DOX | 0.2 µM DOX | 0.5 µM DOX | | CTL | 0.1 µM DOX | 0.2 µM DOX | 0.5 µM DOX |
| 1 | 2 | 3 | 4 | 5 | 6 | 7 | 8 | 9 | 10 |
| --- | --- | --- | --- | --- | --- | --- | --- | --- | --- |
| L | HUV 133 | HUV 134 | HUV 135 | HUV 136 | L | HUV 137 | HUV 138 | HUV 139 | HUV 140 |
| | CTL | 0.1 µM DOX | 0.2 µM DOX | 0.5 µM DOX | | CTL | 0.1 µM DOX | 0.2 µM DOX | 0.5 µM DOX |
p21
(21 kDa)
α-Tubulin
(52 kDa)
Figure S1F
| 1 | 2 | 3 | 4 | 5 | 6 | 7 | 8 | 9 | 10 | 11 | 12 | 13 |
| --- | --- | --- | --- | --- | --- | --- | --- | --- | --- | --- | --- | --- |
| L | HUV 248 | HUV 249 | HUV 250 | HUV 251 | | HUV 252 | HUV 253 | HUB 256 | HUV 257 | HUV 254 | HUV 255 | L |
| | CTL | 0.1 µM DOX | 0.2 µM DOX | 0.5 µM DOX | | CTL | 0.1 µM DOX | 0.2 µM DOX | 0.5 µM DOX | 0.2 µM DOX | 0.5 µM DOX | |
| 1 | 2 | 3 | 4 | 5 | 6 | 7 | 8 | 9 | 10 |
| --- | --- | --- | --- | --- | --- | --- | --- | --- | --- |
| L | HUV 187 | HUV 188 | HUV 189 | HUV 190 | L | HUV 191 | HUV 192 | HUV 193 | HUV 194 |
| | CTL | 0.1 µM DOX | 0.2 µM DOX | 0.5 µM DOX | | CTL | 0.1 µM DOX | 0.2 µM DOX | 0.5 µM DOX |
| 1 | 2 | 3 | 4 | 5 | 6 | 7 | 8 | 9 | 10 |
| --- | --- | --- | --- | --- | --- | --- | --- | --- | --- |
| L | HUV 73 | HUV 74 | HUV 75 | HUV 76 | L | HUV 79 | HUV 80 | HUV 81 | HUV 82 |
| | CTL | 0.1 µM DOX | 0.2 µM DOX | 0.5 µM DOX | | CTL | 0.1 µM DOX | 0.2 µM DOX | 0.5 µM DOX |
Cyclin D1
(38 kDa)
α-Tubulin
(52 kDa)

## Slide 4
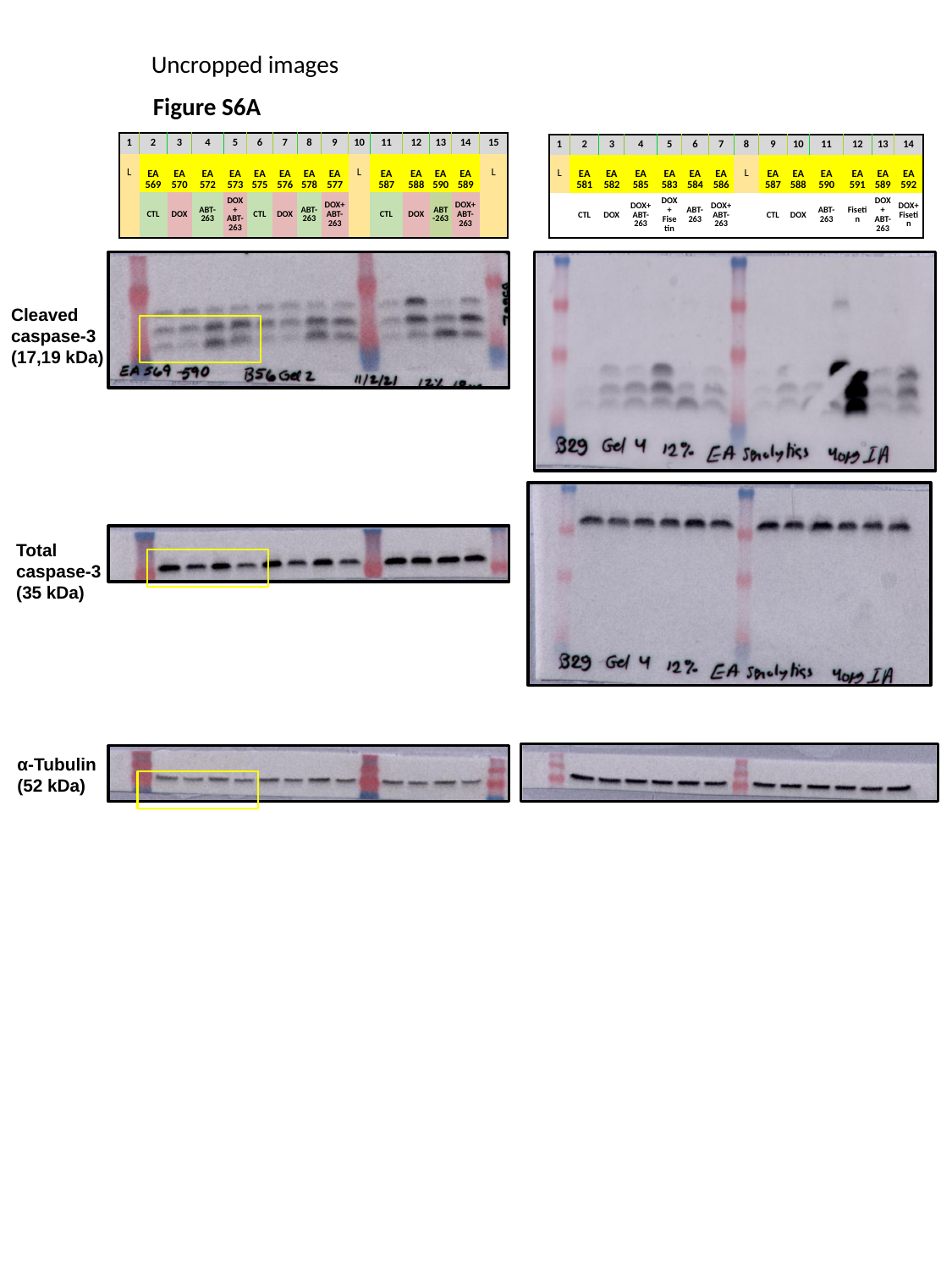

Uncropped images
Figure S6A
| 1 | 2 | 3 | 4 | 5 | 6 | 7 | 8 | 9 | 10 | 11 | 12 | 13 | 14 | 15 |
| --- | --- | --- | --- | --- | --- | --- | --- | --- | --- | --- | --- | --- | --- | --- |
| L | EA 569 | EA 570 | EA 572 | EA 573 | EA 575 | EA 576 | EA 578 | EA 577 | L | EA 587 | EA 588 | EA 590 | EA 589 | L |
| | CTL | DOX | ABT-263 | DOX+ ABT-263 | CTL | DOX | ABT-263 | DOX+ ABT-263 | | CTL | DOX | ABT-263 | DOX+ ABT-263 | |
| 1 | 2 | 3 | 4 | 5 | 6 | 7 | 8 | 9 | 10 | 11 | 12 | 13 | 14 |
| --- | --- | --- | --- | --- | --- | --- | --- | --- | --- | --- | --- | --- | --- |
| L | EA 581 | EA 582 | EA 585 | EA 583 | EA 584 | EA 586 | L | EA 587 | EA 588 | EA 590 | EA 591 | EA 589 | EA 592 |
| | CTL | DOX | DOX+ ABT-263 | DOX+ Fisetin | ABT-263 | DOX+ ABT-263 | | CTL | DOX | ABT-263 | Fisetin | DOX+ ABT-263 | DOX+ Fisetin |
Cleaved caspase-3
(17,19 kDa)
Total caspase-3
(35 kDa)
α-Tubulin
(52 kDa)

## Slide 5
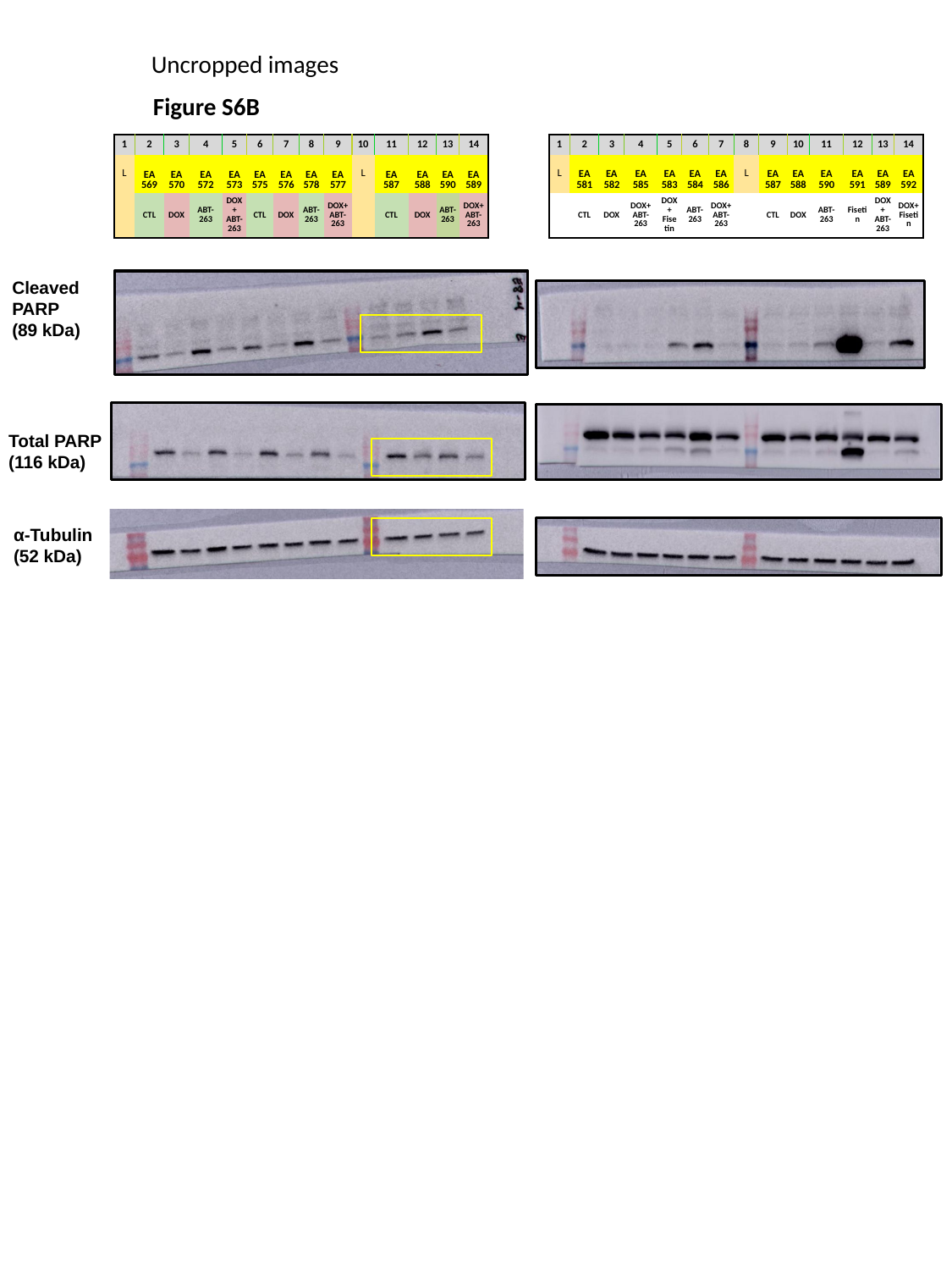

Uncropped images
Figure S6B
| 1 | 2 | 3 | 4 | 5 | 6 | 7 | 8 | 9 | 10 | 11 | 12 | 13 | 14 |
| --- | --- | --- | --- | --- | --- | --- | --- | --- | --- | --- | --- | --- | --- |
| L | EA 569 | EA 570 | EA 572 | EA 573 | EA 575 | EA 576 | EA 578 | EA 577 | L | EA 587 | EA 588 | EA 590 | EA 589 |
| | CTL | DOX | ABT-263 | DOX+ ABT-263 | CTL | DOX | ABT-263 | DOX+ ABT-263 | | CTL | DOX | ABT-263 | DOX+ ABT-263 |
| 1 | 2 | 3 | 4 | 5 | 6 | 7 | 8 | 9 | 10 | 11 | 12 | 13 | 14 |
| --- | --- | --- | --- | --- | --- | --- | --- | --- | --- | --- | --- | --- | --- |
| L | EA 581 | EA 582 | EA 585 | EA 583 | EA 584 | EA 586 | L | EA 587 | EA 588 | EA 590 | EA 591 | EA 589 | EA 592 |
| | CTL | DOX | DOX+ ABT-263 | DOX+ Fisetin | ABT-263 | DOX+ ABT-263 | | CTL | DOX | ABT-263 | Fisetin | DOX+ ABT-263 | DOX+ Fisetin |
Cleaved PARP
(89 kDa)
Total PARP
(116 kDa)
α-Tubulin
(52 kDa)

## Slide 6
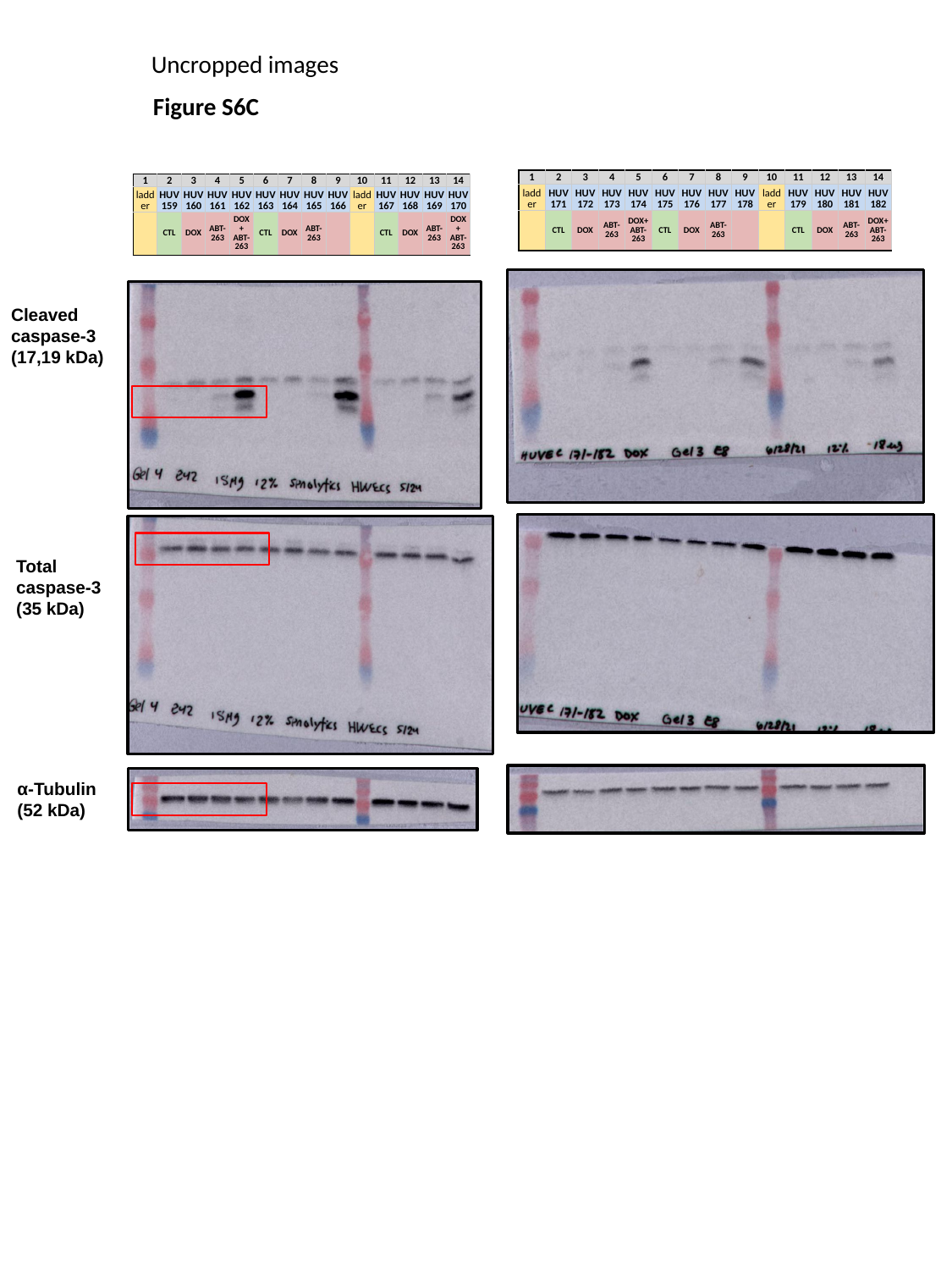

Uncropped images
Figure S6C
| 1 | 2 | 3 | 4 | 5 | 6 | 7 | 8 | 9 | 10 | 11 | 12 | 13 | 14 |
| --- | --- | --- | --- | --- | --- | --- | --- | --- | --- | --- | --- | --- | --- |
| ladder | HUV 171 | HUV 172 | HUV 173 | HUV 174 | HUV 175 | HUV 176 | HUV 177 | HUV 178 | ladder | HUV 179 | HUV 180 | HUV 181 | HUV 182 |
| | CTL | DOX | ABT-263 | DOX+ ABT-263 | CTL | DOX | ABT-263 | | | CTL | DOX | ABT-263 | DOX+ ABT-263 |
| 1 | 2 | 3 | 4 | 5 | 6 | 7 | 8 | 9 | 10 | 11 | 12 | 13 | 14 |
| --- | --- | --- | --- | --- | --- | --- | --- | --- | --- | --- | --- | --- | --- |
| ladder | HUV 159 | HUV 160 | HUV 161 | HUV 162 | HUV 163 | HUV 164 | HUV 165 | HUV 166 | ladder | HUV 167 | HUV 168 | HUV 169 | HUV 170 |
| | CTL | DOX | ABT-263 | DOX+ ABT-263 | CTL | DOX | ABT-263 | | | CTL | DOX | ABT-263 | DOX+ ABT-263 |
Cleaved caspase-3
(17,19 kDa)
Total caspase-3
(35 kDa)
α-Tubulin
(52 kDa)

## Slide 7
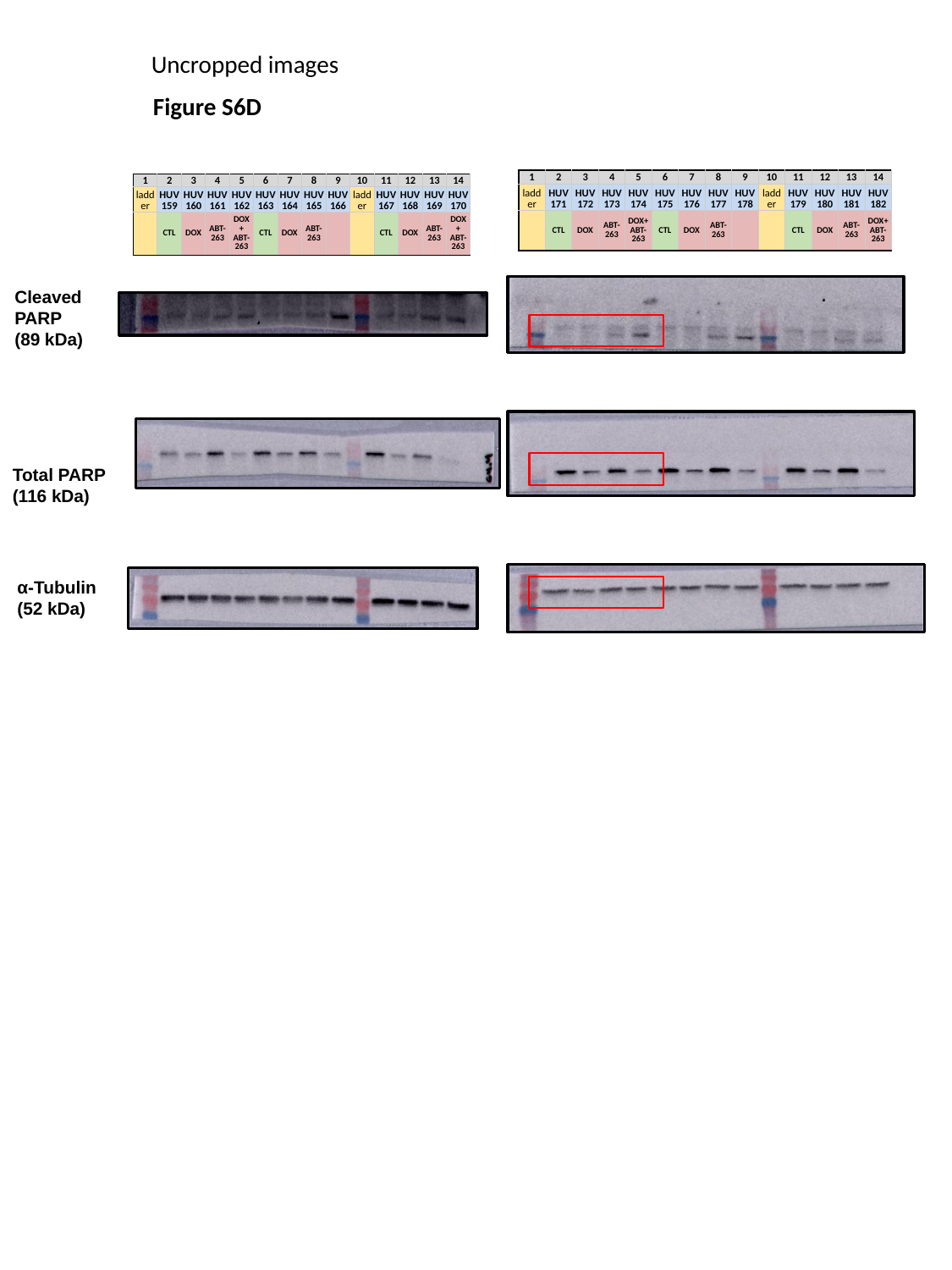

Uncropped images
Figure S6D
| 1 | 2 | 3 | 4 | 5 | 6 | 7 | 8 | 9 | 10 | 11 | 12 | 13 | 14 |
| --- | --- | --- | --- | --- | --- | --- | --- | --- | --- | --- | --- | --- | --- |
| ladder | HUV 171 | HUV 172 | HUV 173 | HUV 174 | HUV 175 | HUV 176 | HUV 177 | HUV 178 | ladder | HUV 179 | HUV 180 | HUV 181 | HUV 182 |
| | CTL | DOX | ABT-263 | DOX+ ABT-263 | CTL | DOX | ABT-263 | | | CTL | DOX | ABT-263 | DOX+ ABT-263 |
| 1 | 2 | 3 | 4 | 5 | 6 | 7 | 8 | 9 | 10 | 11 | 12 | 13 | 14 |
| --- | --- | --- | --- | --- | --- | --- | --- | --- | --- | --- | --- | --- | --- |
| ladder | HUV 159 | HUV 160 | HUV 161 | HUV 162 | HUV 163 | HUV 164 | HUV 165 | HUV 166 | ladder | HUV 167 | HUV 168 | HUV 169 | HUV 170 |
| | CTL | DOX | ABT-263 | DOX+ ABT-263 | CTL | DOX | ABT-263 | | | CTL | DOX | ABT-263 | DOX+ ABT-263 |
Cleaved PARP
(89 kDa)
Total PARP
(116 kDa)
α-Tubulin
(52 kDa)

## Slide 8
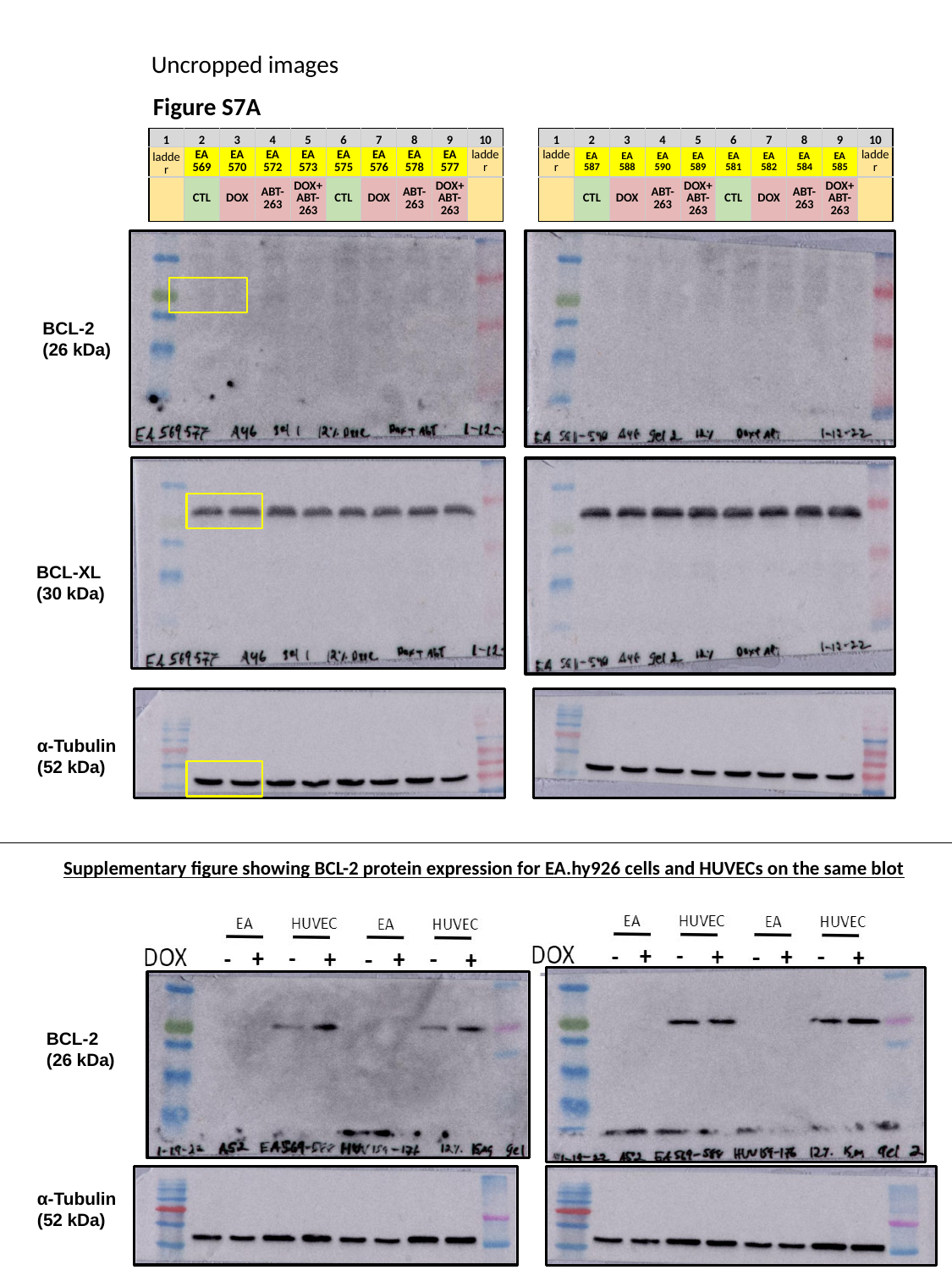

Uncropped images
Figure S7A
| 1 | 2 | 3 | 4 | 5 | 6 | 7 | 8 | 9 | 10 |
| --- | --- | --- | --- | --- | --- | --- | --- | --- | --- |
| ladder | EA 569 | EA 570 | EA 572 | EA 573 | EA 575 | EA 576 | EA 578 | EA 577 | ladder |
| | CTL | DOX | ABT-263 | DOX+ ABT-263 | CTL | DOX | ABT-263 | DOX+ ABT-263 | |
| 1 | 2 | 3 | 4 | 5 | 6 | 7 | 8 | 9 | 10 |
| --- | --- | --- | --- | --- | --- | --- | --- | --- | --- |
| ladder | EA 587 | EA 588 | EA 590 | EA 589 | EA 581 | EA 582 | EA 584 | EA 585 | ladder |
| | CTL | DOX | ABT-263 | DOX+ ABT-263 | CTL | DOX | ABT-263 | DOX+ ABT-263 | |
BCL-2
(26 kDa)
BCL-XL
(30 kDa)
α-Tubulin
(52 kDa)
Supplementary figure showing BCL-2 protein expression for EA.hy926 cells and HUVECs on the same blot
BCL-2
(26 kDa)
α-Tubulin
(52 kDa)

## Slide 9
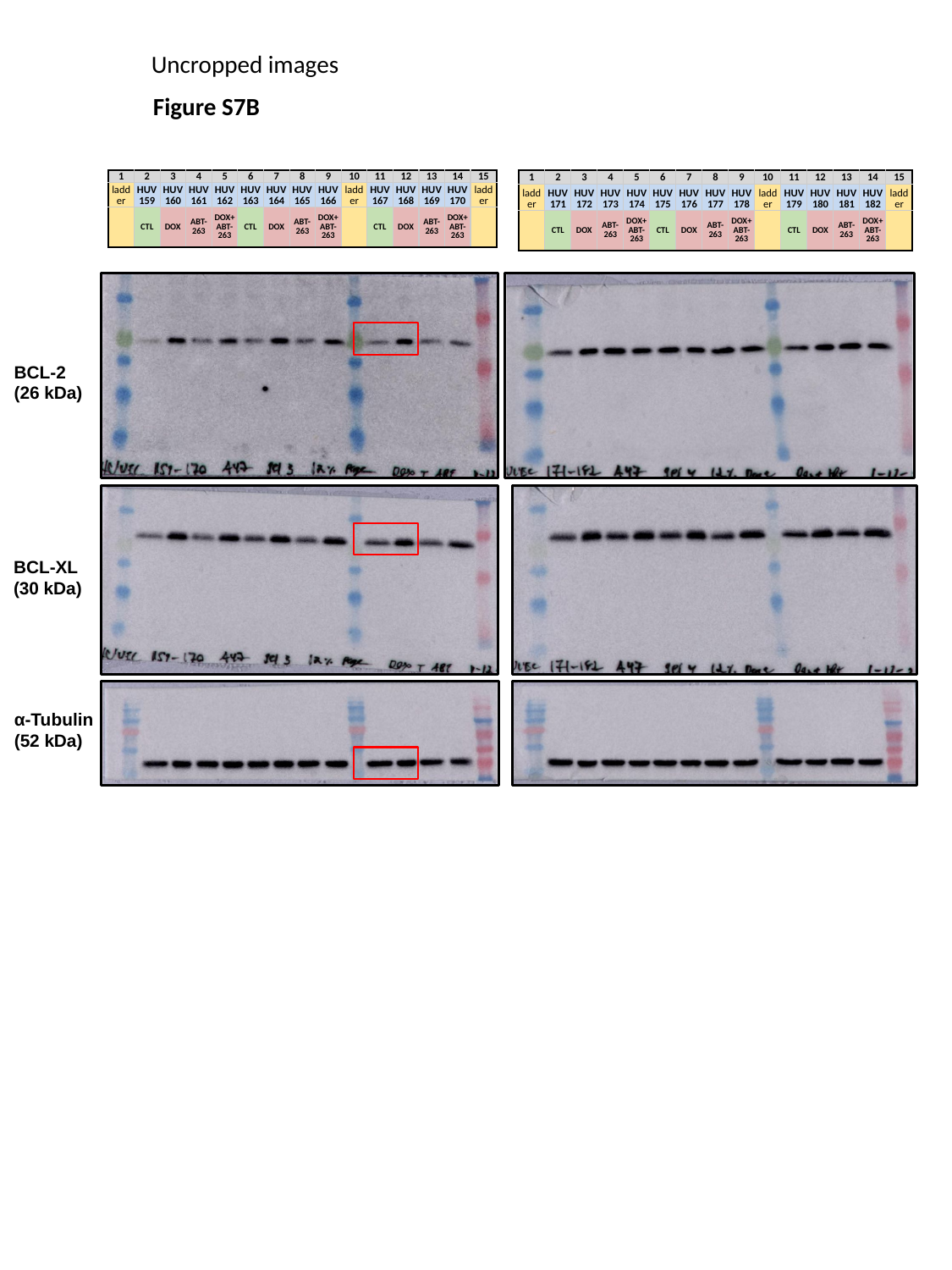

Uncropped images
Figure S7B
| 1 | 2 | 3 | 4 | 5 | 6 | 7 | 8 | 9 | 10 | 11 | 12 | 13 | 14 | 15 |
| --- | --- | --- | --- | --- | --- | --- | --- | --- | --- | --- | --- | --- | --- | --- |
| ladder | HUV 159 | HUV 160 | HUV 161 | HUV 162 | HUV 163 | HUV 164 | HUV 165 | HUV 166 | ladder | HUV 167 | HUV 168 | HUV 169 | HUV 170 | ladder |
| | CTL | DOX | ABT-263 | DOX+ ABT-263 | CTL | DOX | ABT-263 | DOX+ ABT-263 | | CTL | DOX | ABT-263 | DOX+ ABT-263 | |
| 1 | 2 | 3 | 4 | 5 | 6 | 7 | 8 | 9 | 10 | 11 | 12 | 13 | 14 | 15 |
| --- | --- | --- | --- | --- | --- | --- | --- | --- | --- | --- | --- | --- | --- | --- |
| ladder | HUV 171 | HUV 172 | HUV 173 | HUV 174 | HUV 175 | HUV 176 | HUV 177 | HUV 178 | ladder | HUV 179 | HUV 180 | HUV 181 | HUV 182 | ladder |
| | CTL | DOX | ABT-263 | DOX+ ABT-263 | CTL | DOX | ABT-263 | DOX+ ABT-263 | | CTL | DOX | ABT-263 | DOX+ ABT-263 | |
BCL-2
(26 kDa)
BCL-XL
(30 kDa)
α-Tubulin
(52 kDa)

## Slide 10
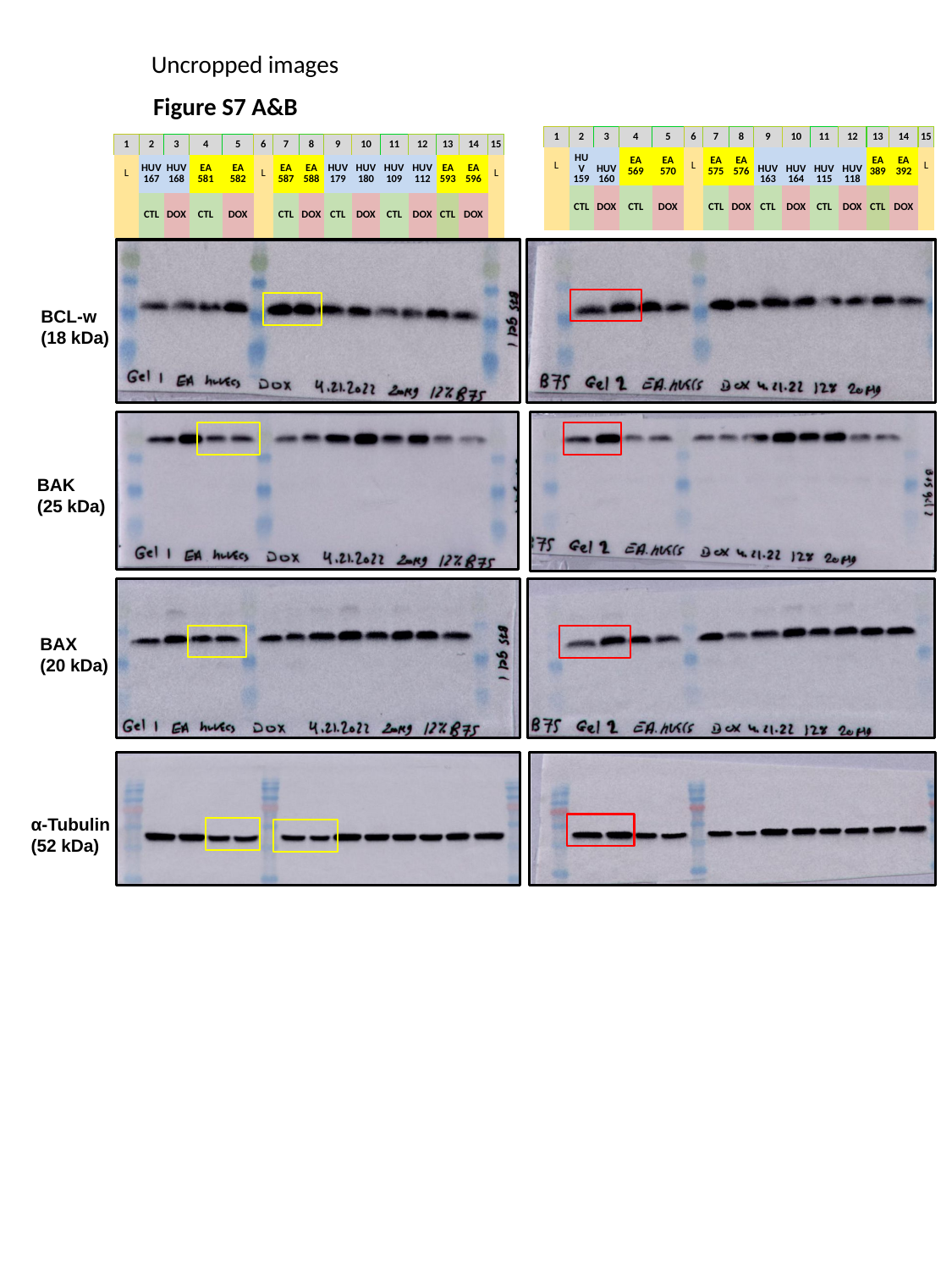

Uncropped images
Figure S7 A&B
| 1 | 2 | 3 | 4 | 5 | 6 | 7 | 8 | 9 | 10 | 11 | 12 | 13 | 14 | 15 |
| --- | --- | --- | --- | --- | --- | --- | --- | --- | --- | --- | --- | --- | --- | --- |
| L | HUV 159 | HUV 160 | EA 569 | EA 570 | L | EA 575 | EA 576 | HUV 163 | HUV 164 | HUV 115 | HUV 118 | EA 389 | EA 392 | L |
| | CTL | DOX | CTL | DOX | | CTL | DOX | CTL | DOX | CTL | DOX | CTL | DOX | |
| 1 | 2 | 3 | 4 | 5 | 6 | 7 | 8 | 9 | 10 | 11 | 12 | 13 | 14 | 15 |
| --- | --- | --- | --- | --- | --- | --- | --- | --- | --- | --- | --- | --- | --- | --- |
| L | HUV 167 | HUV 168 | EA 581 | EA 582 | L | EA 587 | EA 588 | HUV 179 | HUV 180 | HUV 109 | HUV 112 | EA 593 | EA 596 | L |
| | CTL | DOX | CTL | DOX | | CTL | DOX | CTL | DOX | CTL | DOX | CTL | DOX | |
BCL-w
(18 kDa)
BAK
(25 kDa)
BAX
(20 kDa)
α-Tubulin
(52 kDa)
